# Regulated degradation of the APC coactivator Cdc20

**DOI:** 10.1186/1747-1028-5-23

**Published:** 2010-09-10

**Authors:** Jonathan A Robbins, Frederick R Cross

**Affiliations:** 1 Laboratory of Yeast Molecular Genetics, The Rockefeller University, New York, NY, USA

## Abstract

**Background:**

Cdc20 is a highly conserved activator of the anaphase-promoting complex (APC), promoting cell-cycle-regulated ubiquitination and proteolysis of a number of critical cell-cycle-regulatory targets including securin and mitotic cyclins. APC-Cdc20 activity is tightly regulated, and this regulation is likely important for accurate cell cycle control. One significant component of Cdc20 regulation is thought to be Cdc20 proteolysis. However, published literature suggests different mechanisms and requirements for Cdc20 proteolysis. The degree to which Cdc20 proteolysis is cell-cycle regulated, the dependence of Cdc20 proteolysis on Cdc20 destruction boxes (recognition sequences for APC-mediated ubiqutination, either by Cdc20 or by the related Cdh1 APC activator), and the need for APC itself for Cdc20 proteolysis all have been disputed to varying extents. In animals, Cdc20 proteolysis is thought to be mediated by Cdh1, contributing an intrinsic order of APC activation by Cdc20 and then by Cdh1. One report suggests a Cdh1 requirement for Cdc20 proteolysis in budding yeast; this idea has not been tested further.

**Results:**

We characterized Cdc20 proteolysis using Cdc20 expressed from its endogenous locus; previous studies generally employed strongly overexpressed Cdc20, which can cause significant artifacts. We analyzed Cdc20 proteolysis with or without mutations in previously identified destruction box sequences, using varying methods of cell cycle synchronization, and in the presence or absence of Cdh1. Cdc20 instability is only partially dependent on destruction boxes. A much stronger dependence on Cdh1 for Cdc20 proteolysis was observed, but Cdh1-independent proteolysis was also clearly observed. Cdc20 proteolysis independent of both destruction boxes and Cdh1 was especially detectable around the G1/S transition; Cdh1-dependent proteolysis was most notable in late mitosis and G1.

**Conclusions:**

Cdc20 proteolysis is under complex control, with different systems operating at different points in the cell cycle. This complexity is likely to explain apparent conflicts in previously published literature on this subject. A major mode of control of Cdc20 proteolysis occurs in late mitosis/early G1 and is Cdh1-dependent, as in animal cells; this mode may contribute to the known sequential activation of the APC by Cdc20 followed by Cdh1. An independent mode of Cdc20 proteolysis, independent of destruction boxes and Cdh1, occurs at G1/S; we do not know the mechanism or function of this mode of proteolysis, but speculate that it may contribute to sharpening and restricting activation of APC-Cdc20 to early mitosis.

## Background

The oscillation of cyclin dependent kinase (CDK) activity lies at the heart of the cell cycle, serving to coordinate the events of the cell cycle in a temporally appropriate manner. CDK activity is dependent upon CDK binding to a partner cyclin [1]; to exit from mitosis, the CDK activity of the mitotic B-type cyclins must be reduced; this occurs largely by cyclin destruction. The anaphase-promoting complex (APC) is a ubiquitin ligase responsible for the destruction of cyclins at the end of mitosis: the cell cycle ends in highly efficient and specific protein destruction orchestrated by the APC, which mediates the sequential degradation of cyclins and other relevant cell cycle proteins and machinery [2,3].

The APC is a large ubiquitin E3 ligase comprised of at least 13 proteins, and functions in coordination with two homologous mitotic coactivators, Cdc20p and Cdh1 [4-8]. The APC and both coactivators are conserved throughout eukaryotic evolution. The APC is active only from anaphase onset through the subsequent G1, although the core complex is present throughout the cell cycle. The conserved coactivators Cdc20 and Cdh1 provide regulation of timing and specificity. APC-Cdc20 begins B-type cyclin degradation and APC-Cdh1 continues it through mitosis and into the ensuing G1 [5,9-13].

A major basis for this difference in timing is differential regulation of APC-Cdc20 and APC-Cdh1 by cyclin-CDK activity. APC-Cdc20 is active at high CDK levels, with Cdc20 binding preferentially to CDK-phosphorylated APC [14,15]. Cdc20 itself is an unstable protein, accumulating late in the cell cycle, followed by mitotic degradation [16-18]. As B-type cyclin levels decline and the Cdc14 phosphatase (at least in budding yeast) is released from a nucleolar sequestration, the balance between CDK activity and phosphatase activity shifts such that Cdh1 is dephosphorylated on at least some of its 11 CDK sites, which collectively serve to inhibit Cdh1 function [10]. The second wave of APC-mediated degradation then ensues, dependent on dephosphorylated Cdh1. This activity is responsible for continued mitotic cyclin degradation through G1, until Cdh1 inactivation in the succeeding cell cycle [19].

In addition to these temporal differences, Cdc20 and Cdh1 likely have intrinsically different substrate specificities, although they both contribute to mitotic cyclin degradation. Cdc20 promotes Pds1 proteolysis, an anaphase inhibitor that prevents cleavage of cohesin, the protein keeping sister chromatids attached [11,20]. APC-Cdh1 seems ineffective at promoting Pds1 degradation, but promotes degradation of several spindle proteins and perhaps Cdc20 itself [5,10,16,21-24]. This ordering is logical: the earlier APC-Cdc20 wave will promote anaphase and initial mitotic cyclin proteolysis, promoting APC-Cdh1 activation; APC-Cdh1 then completes mitotic cyclin proteolysis, allowing cytokinesis and other events of mitotic exit, removes Cdc20 to reset the system to G1, and contributes to spindle disassembly by proteolysis of spindle components. This ordering could help ensure that anaphase precedes cytokinesis and spindle disassembly.

Specific motifs in substrate proteins target them for APC-mediated ubiquitination: the destruction box (consensus RxxL) [25], recognized by Cdc20 and Cdh1; the KEN box, which may be more specific for Cdh1 [26] (but see [27]), and the CRY box Cdh1 recognition sequence [28]. Thus Cdh1 recognizes unique motifs that Cdc20 does not; in contrast, there are no known Cdc20-specific targeting sequences, although Cdc20 specific substrates exist.

*CDC20 *is essential for cell viability, and its absence results in an arrest with unseparated sister chromatids and high Clb2 levels [16,29]. Deletion of the APC-Cdc20 target *PDS1 *(securin) allows *cdc20 *cells to undergo anaphase [16,29]. Further deletion of *CLB5 *results in a viable *cdc20 pds1 clb5 *triple mutant, capable of carrying out all essential cell-cycle functions [11]. This defines two critical targets of Cdc20; consistently, both have been reported to be poor APC-Cdh1 substrates [5,6].

Temporal separation of APC-Cdc20 and APC-Cdh1 activity is thought to promote ordering of degradation of APC substrates. Not only must Cdh1 activity be restrained until mitotic exit, but it is likely that Cdc20 must be inactivated for the subsequent cell cycle. Inability to inactivate Cdc20 would impede securin accumulation, impairing separase regulation, and constitutive Cdc20 could also block accumulation of the major S-phase cyclin Clb5.

Three mechanisms are known to contribute to Cdc20 inactivation: the dephosphorylation of the APC [14,15], transcriptional shutoff [17], and the destruction of Cdc20 itself [16-18,22,30]. Cdc20 has two destruction boxes thought to target it for destruction [16]. It has been argued based on the stabilization of overexpressed alleles deleted for the region containing destruction boxes that both destruction boxes contribute to Cdc20 degradation in G1 [16,17]. One study also found residual APC-dependent but destruction box independent Cdc20 instability throughout the entire cell cycle [17]. Another study argued that degradation of Cdc20 was dependent only on the first destruction box [30], and was cell-cycle-regulated (highest in G1), but was independent of APC activity. These substantially contradictory results hamper understanding of this potentially important regulatory event.

## Results

### The role of the Cdc20 destruction boxes

**Figure 1:**
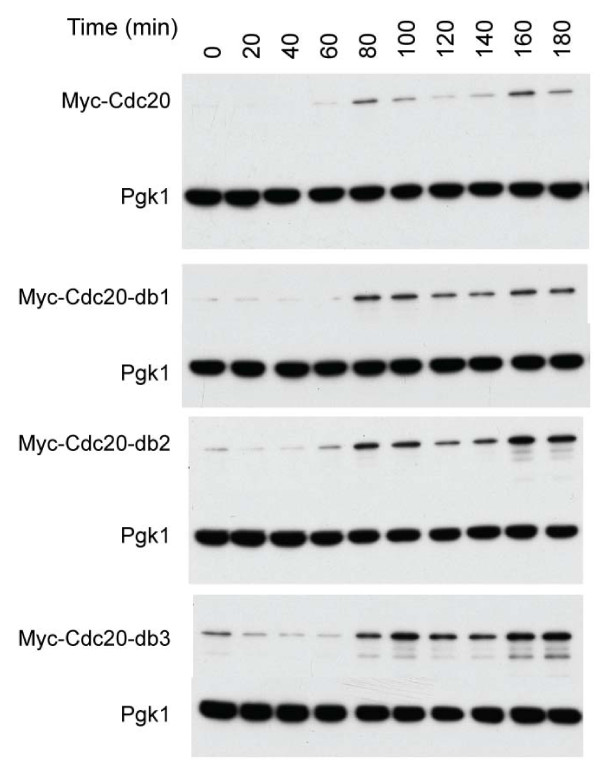
**Destruction boxes contribute to, but are not solely responsible for, the destruction of Cdc20**. Strains bearing *18MYC*-tagged *CDC20 *with either destruction box 1 (db1), destruction box 2 (db2), or both destruction boxes (db3) ablated were synchronized with alpha-factor and released. Immunoblots against Myc are shown, with Pgk1 serving as a loading control.

**Figure 2:**
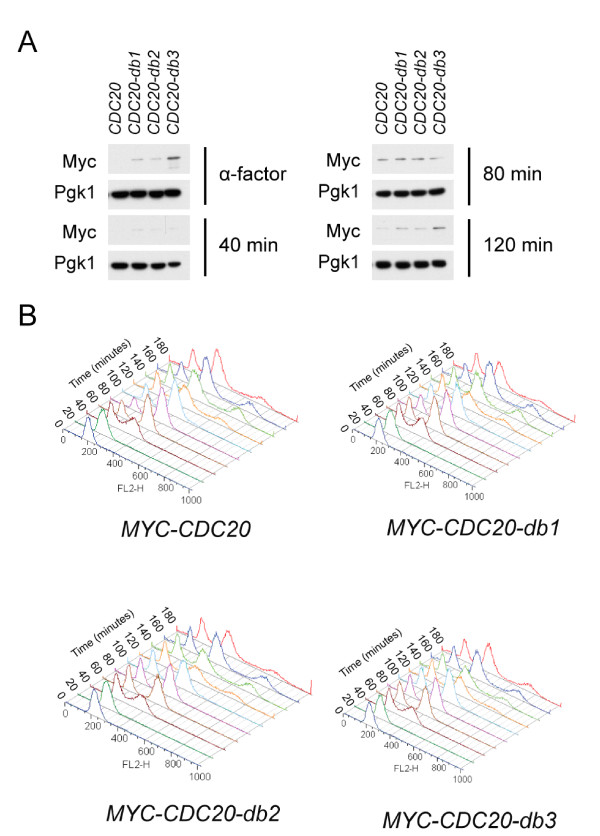
**Ablation of destruction boxes particularly stabilizes Cdc20 in alpha-factor, but does not affect DNA replication kinetics**. **A **Samples from Figure 1 for each *CDC20 *allele at indicated timepoints were loaded next to one another on the same gel and blotted against. **B **FACS analysis of DNA content from samples taken in parallel with those from Figure 1.

We sought to test the consequences of removing the individual Cdc20 destruction boxes, in the context of the endogenous locus and promoter, using the N-terminally myc-epitope-tagged construct described by [16]. The destruction box mutations were alterations of the RxxL consensus site to AxxA. *CDC20-db1 *lacks the first destruction box, *CDC20-db2 *the second, and *CDC20-db3 *both (nomenclature of [16]). These alleles were functional, as they replaced the endogenous copy of *CDC20*, which is an essential gene. Additionally, they were also viable in the absence of *CDH1*, a genetic background known to be sensitive to hypoactive APC-Cdc20 [31]. Cells were synchronized in alpha-factor and released, with Myc-Cdc20 accumulation monitored by western blotting against Myc (Figure 1). Wild-type Cdc20 was completely gone at the alpha-factor block; this clearance required the two destruction boxes (Figure 2A). Upon release from alpha-factor, Cdc20 accumulated strikingly, with a second drop and reaccumulation detectable (presumably due to mitosis and cell cycle reentry). While the db mutations resulted in increased Cdc20 levels and less efficient clearance in mitosis, even Cdc20-db3 retained a pattern of accumulation generally similar to wild-type.

We noted a reproducible decrease in the db-mutated Cdc20 proteins upon release of the alpha-factor block. (A similar effect was detectable upon long exposure with wild-type Cdc20, despite the extremely low initial levels; data not shown.) *CDC20 *transcription is low at alpha-factor arrest, and only increases late in the cell cycle [32]; thus, the decline in Cdc20 levels shortly after release from the alpha-factor block is probably not the consequence of transcriptional downregulation. Rather, Cdc20 appears to be actively destroyed by some process as cells progress through G1 into S. This degradation is destruction box-independent, and is unlikely to be APC-Cdh1 mediated, as Cdh1 activity is very high in alpha-factor-blocked cells and declines to negligible levels upon release.

Overexpression of *CDC20 *lacking destruction boxes has been reported to interfere with S-phase progression (perhaps due to APC-Cdc20 ubiquitination of the S-phase cyclin Clb5) [11]. However, with *CDC20-db3 *expressed at endogenous levels we observe at most minor effects on DNA replication kinetics (Figure 2B).

### Overexpression of *CDH1 *partially reduces Cdc20 levels

**Figure 3:**
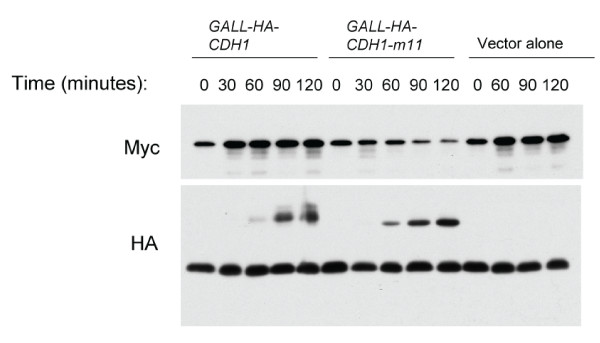
**Overexpressed *CDH1-m11 *lowers Cdc20 levels, but does not clear it**. Either *HA-CDH1 *or *HA-CDH1-m11 *was induced using deoxycorticosterone in cycling strains with a GAL4-Mineralocorticoid receptor fusion (containing the DNA binding domain of the former, and ligand binding domain of the latter, rendering GAL responsive genes inducible by exogenous mineralocorticoids [38]). Levels of endogenously expressed 18MYC-Cdc20 and HA-Cdh1 were followed by immunoblot, with a nonspecific band reactive with the anti-HA antibody serving as a loading control.

APC-Cdh1 activity has been reported to restrain Cdc20 accumulation until early S-phase [22]. Cdc20 was nearly completely removed in alpha-factor-blocked cells, even in the absence of its destruction boxes. Alpha-factor-blocked cells contain high APC-Cdh1 activity, which could account for Cdc20 clearance in these cells. Therefore, we tested whether Cdh1 overexpression in cycling cells is able to clear Cdc20 expressed from the endogenous locus. Overexpressed *CDH1 *had little effect on Cdc20 levels, but this could be due to efficient Cdk phosphorylation and inactivation of Cdh1 (Figure 3). *CDH1-m11*, lacking Cdk sites, reduces the level of Cdc20 significantly; however, this effect was incomplete even after two hours of *CDH1-m11 *induction (Figure 3), a time long enough for Cdh1-m11 to induce cell cycle arrest with a hyperpolarized morphology characteristic of an absence of B-type cyclins (data not shown). This induction results in significant overexpression as compared to expression from the endogenous promoter ([10]; data not shown). It is unclear why overexpressed constitutively active Cdh1 is unable to completely clear Cdc20, which is in contrast to the complete clearance seen in alpha-factor blocked cells, or in *CDH1-m11 *(exact gene replacement) cells released from alpha-factor and accumulating at the *CDH1-m11 *block[33]. As this is an initially asynchronous culture, and *CDH1-m11 *expressing cells arrest in the first cycle, it possible that during this arrest Cdc20 is a poor Cdh1 substrate, perhaps owing to posttranslational modifications, association with the APC or spindle checkpoint proteins, or spatial sequestration of Cdc20. It is also possible that *CDC20 *transcription at this block is sufficient to reach a steady-state equilibrium.

### Cdc20 levels are increased but still cell cycle regulated in the absence of *CDH1*

**Figure 4:**
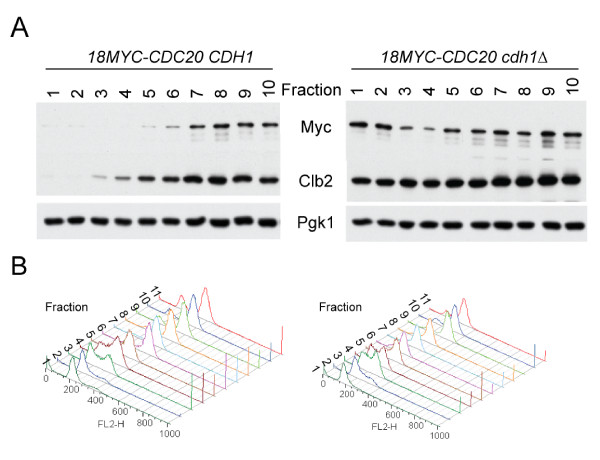
**Deletion of *CDH1 *partially stabilizes Cdc20**. **A ***CDH1 18MYC-CDC20 *and *cdh1 18MYC-CDC20 *strains were elutriated, and the resultant fractions were immunoblotted for Myc and Clb2. Pgk1 serves as a loading control. **B **FACS to assess DNA content for the fractions collected in (A).

To examine the effect of endogenous Cdh1 on Cdc20 levels, we used centrifugal elutriation to separate cycling *cdh1 *and wild-type cells into different cell cycle fractions based on cell size (we were unable to use alpha-factor synchronization for this experiment because *cdh1 *cells do not arrest properly in response to alpha-factor [5]. The use of size fractionation followed by direct analysis of different-sized cell fractions has been validated previously for wild-type and *cdh1 *mutants [34]). *cdh1 *cells have higher levels of Cdc20 than *CDH1 *cells, particularly in G1; however, Cdc20 declines to a low level in S-phase *cdh1 *cells (Figure 4), before increasing later in the cell cycle. Thus, Cdh1 may be responsible for clearance of Cdc20 in G1, but there appear to be Cdh1-independent mechanisms of Cdc20 clearance operating later in the cell cycle. These observations are consistent with those made above with alpha-factor synchronized *CDC20-db3 *cells, and with *CDH1-m11-*overexpressing cells.

### Inducible Cdc20 is degraded by both Cdh1-dependent and independent mechanisms

**Figure 5:**
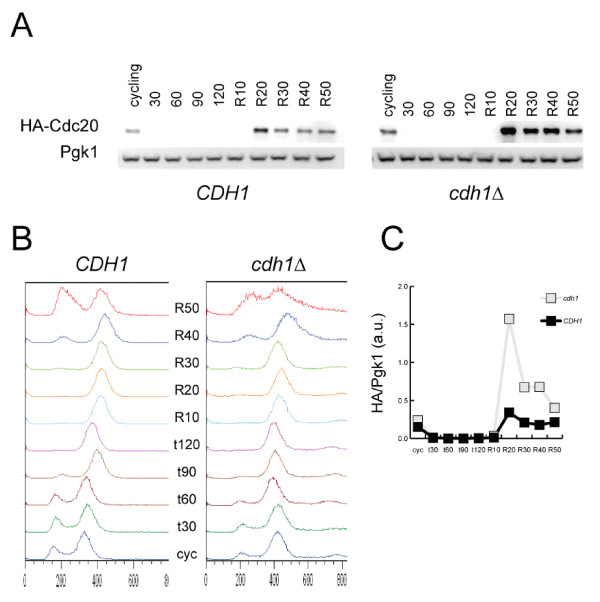
**Inducible Cdc20 is degraded by both Cdh1 dependent and independent mechanisms**. **A **Methionine was added at time 0 to cycling *CDH1 *and *cdh1 *strains, with *MET3pr-HA-CDC20 *(methionine repressible) replacing endogenous *CDC20*, to shutoff *CDC20 *transcription. After two hours, these cells were released into methionine-free medium, inducing *CDC20 *transcription. Samples were taken every ten minutes thereafter (R10, R20, etc). HA-Cdc20 was immunoblotted against, with Pgk1 serving as a loading control. **B **DNA content of the strains in (A) was assessed by FACS. **C **Quantification of immunoblots in (A).

To pursue Cdh1-dependent and-independent mechanisms of Cdc20 proteolysis, independent of transcriptional regulation of *CDC20*, we employed an inducible *MET3-HA-CDC20 *replacing the endogenous copy of *CDC20*. Depletion of Cdc20 in this strain by methionine addition results in a reversible metaphase arrest. *MET3-HA-CDC20 CDH1 *and *MET3-HA-CDC20 cdh1 *strains were grown to log phase in medium lacking methionine. Methionine was then added; 30 minutes later, neither strain contained detectable Cdc20, once again confirming the existence of efficient Cdh1-independent mechanisms of Cdc20 degradation (Figure 5). Upon induction of *CDC20 *to release the metaphase block, Cdc20 levels increase much more in a *cdh1 *than in a *CDH1 *background, confirming the ability of Cdh1 to promote Cdc20 degradation during mitotic exit. However, after a short additional time Cdc20 levels decrease substantially in the *cdh1 *cells as well (although the levels remain higher than in *CDH1 *cells) (Figure 5).

These results confirm the existence of separable Cdh1-dependent and-independent mechanisms of Cdc20 proteolysis.

## Discussion

The regulatory control of Cdc20 has implications for the proper ordering of cell cycle events. Multiple mechanisms are involved in its regulation including transcriptional control, Cdc20 protein destruction, and CDK phosphorylation of the APC. Both *CDC20 *transcriptional control and CDK phosphorylation of Cdc16, Cdc23, and Cdc27 APC components are dispensable for cell cycle progression [13,14]. Cdc20 proteolysis provides a third control mechanism, but the literature is unclear as to how Cdc20 proteolysis is controlled. In this work, we attempt to clarify the agents, timing and motifs involved in Cdc20 destruction.

Conflicting reports exist in the literature as to what mediates the destruction of Cdc20. Studies have variously implicated the APC, probably independent of Cdh1 because of the timing of proteolysis [16,17], APC-Cdh1 specifically [22], and an APC-independent mechanism [30]. Here we find evidence for both APC-Cdh1 dependent and independent mechanisms, operating at different times in the cell cycle; the existence of multiple mechanisms may account for the conflicting nature of previous reports. One caveat to our experiments are the use of epitope tags, for which there always exists a potential for tag artifacts to occur. However, we find consistent evidence for APC dependent and independent mechanisms of Cdc20 degradation using both Myc and HA tags, arguing against the presence of such an artifact. Additionally, the epitope tags have the advantage of precluding the possibility that the antibody is affected by either posttranslational modifications or constructed mutations of Cdc20 (such as destruction box ablation-we have found that the destruction box and KEN boxes of the Clb2 mitotic cyclin can be major epitopes for rabbit antibodies, for example [B. Drapkin, pers. comm.]).

We find that APC-Cdh1 makes the major contribution to Cdc20 proteolysis from mitotic exit through the subsequent G1 (Figure 4). This provides a simple mechanism for temporal separation of APC-Cdc20 and APC-Cdh1 activity: as Cdh1 is activated in late mitosis, Cdc20 is effectively removed by Cdh1-dependent proteolysis. Beginning in late G1/early S, a Cdh1-independent mechanism can also carry out Cdc20 degradation. We do not know what proteins control this mechanism, preventing further analysis. The efficiency of removal of destruction box-containing Cdc20 by Cdh1 suggests that this second mechanism is not of great importance for regulating Cdc20 levels. We speculate that this may play role in limiting Cdc20 accumulation prior to anaphase so as to prevent premature cohesin cleavage, or may contribute to efficient engagement of the spindle checkpoint during aberrant mitoses (since Cdc20 is the ultimate target of this checkpoint).

APC-dependent but destruction box independent degradation of Cdc20 was reported in S phase and mitosis [17]; it is possible that this is the Cdh1-and destruction box-independent Cdc20 degradation we observe. Cdh1 would not be expected to be active in these phases of the cell cycle, and thus any APC-mediated degradation of Cdc20 would likely result from a direct APC Cdc20 interaction (especially as characterized APC-Cdc20 substrates are stable at this time). We have not evaluated the role of APC-dependent but coactivator-independent ubiquitination of Cdc20.

Work based upon overexpression studies of *CDC20 *alleles with destruction box deletions has arrived at conflicting conclusions for the relative contributions of the two destruction boxes [16,17,30]. We find both destruction boxes to contribute to Cdc20 instability, particularly during G1. However, the stabilization conferred by destruction box ablation appears to be considerably weaker than the stabilization due to *CDH1 *removal. This suggests that APC-Cdh1 mediates the destruction of Cdc20 through both destruction box dependent and independent mechanisms. This destruction box independent mechanism could either be through direct degradation or an indirect mechanism such as altering synthesis or localization. There could be additional APC-Cdh1-targeting motifs in Cdc20; one possible targeting motif is a potential KEN box in the C-terminal portion of Cdc20. It is also possible that Cdh1 affects Cdc20 transcription, perhaps by targeting Clb2 which promotes *CDC20 *transcription [35,36].

## Conclusions

Cdc20 proteolysis is under complex control, with different systems operating at different points in the cell cycle. This complexity is likely to explain apparent conflicts in previously published literature on this subject. A major mode of control of Cdc20 proteolysis occurs in late mitosis/early G1 and is Cdh1-dependent, as in animal cells; this mode may contribute to the known sequential activation of the APC by Cdc20 followed by Cdh1. An independent mode of Cdc20 proteolysis, independent of destruction boxes and Cdh1, occurs at G1/S; we do not know the mechanism or function of this mode of proteolysis, but speculate that it may contribute to sharpening and restricting activation of Cdc20-APC to early mitosis.

## Methods

### Yeast strains and plasmids

Standard methods were used throughout. All strains are W303. See Table 1 for strains used. See Table 2 for plasmids used. *CDC20 *mutagenesis was performed by Quickchange Multi-Site Directed Mutagenesis (Stratagene) using the following primers:

Cdc20-db1: AATGCAGCAATTAGCGGTAACgcTTCTGTAgcTTCTATTGCGTCCCCAACAAAGC (creating R17A and L20A)

Cdc20-db2: CTGAACATTAGAAACTCCAAAgcTCCCAGTgcACAAGCCTCTGCCAATTCTATT (creating (R60A and L63A)

**Table 1:** Strains used in this study.

Strain Name	Genotype
JR90	*MATa TRP1-18MYC-CDC20-WT ADE2 URA3::ura3 pRS313:GAL4-MR-HIS3*

JR91	*MATa TRP1-18MYC-CDC20-WT ADE2 GALL-HA-CDH1-WT-URA3::ura3 pRS313:GAL4-MR-HIS3*

JR13	*MATa HIS3::GFP-TUB1 MYO1-GFP-KANMX bar1 MYC-CDC20-TRP1 ADE2*

JR52	*MATa HIS3::GFP-TUB1 MYO1-GFP-KAN bar1 TRP1-18MYC-CDC20-db1 ADE2*

JR55	*MATa HIS3::GFP-TUB1 MYO1-GFP-KANMX bar1 MYC-CDC20-db2-TRP1 ADE2*

JR53	*MATa HIS3::GFP-TUB1 MYO1-GFP-KAN bar1 TRP1-18MYC-CDC20-db3 ADE2*

**Table 2:** Plasmids used in this study.

Plasmid Name	Description	Plasmid Notes
DJC235	*18MYC-CDC20-TRP1*	cut with MluI integrate at CDC20

JRP1	*18MYC-CDC20-db1-TRP1*	cut with MluI to integrate at CDC20

JRP2	*18MYC-CDC20-db2-TRP1*	cut with MluI to integrate at CDC20

JRP3	*18MYC-CDC20-db3-TRP1*	cut with MluI to integrate at CDC20

JRP5	pRS406 *GALL-HA-CDH1*	cut with BglII to integrate at CDH1

JRP6	pRS406 *GALL-HA-CDH1-m11*	cut with BglII to integrate at CDH1

### Synchronization

Alpha-factor time courses were performed by blocking for between two and three hours in alpha-factor, washing 3 × in alpha-factor-free medium, and releasing into the indicated culture conditions.

*MET3-HA-CDC20 *time courses were performed by adding 0.2g/L methionine to methionine-free synthetic medium, arresting for between two and three hours, filtering onto nitrocellulose membranes, washing, and releasing into methionine-free medium.

Centrifugal elutriations were performed using 1L of log phase culture with a Beckman JE5.0 elutriator rotor, running at 3000RPM with sequential fractions elutriated off by stepwise increase in pump speed.

### Immunoblots

Western Blots were performed using standard methods. Antibody concentrations used were: anti-Pgk1 1:10,000 (Invitrogen), anti-HA 12CA5 1:1,000 (Roche), rabbit polyclonal anti-Clb2 1:10,000, Myc 9E10 1:1,000 (Santa Cruz Biotechnology), Clb5 yN-19 (Santa Cruz), Cdc5 yC-19 (Santa Cruz), and HRP-conjugated secondary antibodies at 1:4,000. ECL signal was measured in a Fujifilm DarkBox with CCD camera, and quantified using MultiGauge software (Fujifilm).

### Flow Cytometry

DNA content was assessed through propidium iodide staining of ribonuclease treated cells on a FACSCalibur machine (BD Biosciences), as described [37].

## Competing interests

The authors declare that they have no competing interests.

## Authors' contributions

JAR and FRC designed experiments and drafted the manuscript. JAR performed experiments. Both authors read and approved the final manuscript.
